# Use of CRISPR/Cas9 technology efficiently targetted goat myostatin through zygotes microinjection resulting in double-muscled phenotype in goats

**DOI:** 10.1042/BSR20180742

**Published:** 2018-11-14

**Authors:** Zhengyi He, Ting Zhang, Lei Jiang, Minya Zhou, Daijin Wu, Junyan Mei, Yong Cheng

**Affiliations:** College of Veterinary Medicine/Jiangsu Co-innovation Center for Prevention and Control of Important Animal Infectious Diseases and Zoonosis, Yangzhou University, Yangzhou, Jiangsu 225009, China

**Keywords:** cystine-knot structure, CRISPR/Cas9, genetic stability, health status, MSTN gene

## Abstract

Myostatin gene (*MSTN*) can inhibit the proliferation of myoblast, which in turn promotes muscle growth and inhibits adipocyte differentiation in livestock. MSTN mutation may lead to muscle hypertrophy or double-muscled (DM) phenotype. MSTN mutation animal, such as sheep, dog, and rabbit have been generated through CRISPR/Cas9 technology. However, goats with promising MSTN mutation have not been generated. We designed two sgRNAs loci targetting exon3 of *MSTN* gene to destroy the MSTN cysteines knots. We got seven goats from seven recipients, in which six were MSTN knocked-out (KO) goats, with a mutation rate of 85.7%. Destroyed cysteine knots caused MSTN structure inactivation. The average body weight gain (BWG) per day of MSTN KO goats was significantly higher than that of wild-type (WT) goats. MSTN KO goats showed abnormal sugar, fat, and protein metabolism compared with wild-type controls (MSTN^+/+^). Inheritance of mutations was observed in offspring of MSTN KO goats by PCR analysis.

## Introduction

Myostatin gene (*MSTN*) as a member of transforming growth factor β (TGF-β) family is also called the growth differentiation factor 8 (GDF-8) [1]. MSTN protein is expressed in almost all types of tissues, and the expression level is relatively high in skeletal muscle, which facilitates its role as a negative regulatory factor in the proliferation of myoblast [2]. In contrast, down-regulation of MSTN expression in adult mice not only increases skeletal muscle mass but also increases skeletal muscle strength [3], providing a novel way to improve meat production.

Goats are promising models for genetic modification due to the human-like anatomy, metabolism, physiology, genetics, and size, and goats with MSTN mutation may produce more meat. CRISPR/Cas9 system as a novel genetic modification technique adapted from the adaptive immune system of bacteria and archaea is based in small RNAs and Cas endonucleases proteins that can induce site-specific DNA double-stranded breaks [4]. With the advantages of simple operation, low cost, low off-target rate and high efficiency, CRISPR/Cas9 has been widely used for different purposes, such as disease model construction [5], genetic screens [6], gene function characterization [7], and so on. At present, CRISPR/Cas9 has also been used to generate MSTN mutation animals, such as rabbit [8] and sheep [9]. Construction of FGF5 and MSTN double knockout goat mutant by CRISPR/Cas9 technology has been reported by a previous study [10]. However, no ideal phenotypes of *MSTN* gene knockout have been observed and the correlations between MSTN target loci and targetting efficacy remains unclear. The present study was carried out to solve those problems. In the present study, exon 3 of *MSTN* gene, which has not been targetted before, was selected as the target site to generate MSTN mutation goats using CRISPR/Cas9 technology. The mutation rate, muscle mass yield, genetic stability, and animal health status were discussed.

## Materials and methods

### Ethics statement

Yangtze River Delta white goats were from and maintained at the Research Farm of Yangzhou University, China. All experiments in the present study were performed according to the Guiding Principles for the Care and Use of Laboratory Animals. The present study was approved by the Institutional Animal Care and Use Committee of Yangzhou University, Ministry of Science and Technology of the People’s Republic of China (SYXK2016-0019). All goats were fed the same standard diet and raised under the same conditions. All surgeries were performed under strict aseptic conditions.

### Generation of co-expressing Cas9 and sgRNA plasmid and *in vitro* transcription

To generate vector expressing Cas9 and sgRNA for targetting myostatin exon 3, we designed two sgRNAs named sgRNA-1 and sgRNA-2 to target goat *MSTN* gene using online software (http://crispr.mit.edu) in goats. CRISPR/Cas9 compound plasmid was provided by Nanjing YaoShunYu Biological Technology Co., Ltd (China). See Table 1 for details.

**Table 1:** Sequences and targetting site of sgRNAs

sgRNAs	Sequences	PAM	Targetting sites
sgRNA-1	ATCTTTGTAGGAGTACAGCA	AGG	1012–1034
sgRNA-2	GCATGGTAGTAGATCGCTGT	GGG	1097–1119

PAM means protospacer adjacent motif.

### CRISPR/Cas9 sgRNA and Cas9 *in vitro* transcription

The pYSY-T7-MSTN-gRNA1 and pYSY-T7-MSTN-gRNA2 vectors were *in vitro* transcribed to mRNA and purified using ScriptMAX Thermo T7 Transcription Kit (TSK-101) according to instructions of the kit.

### Zygotes injection with Cas9/sgRNA

Zygotes were collected through surgical oviduct flushing from donors after superovulation treatment and natural mating. Goat embryos at the pronuclear stage (approximately 32–36 h post-mated) were transferred into oocyte manipulation medium, M16 (M7292 – 100 ml, Sigma); M2 (M7167 – 50 ml, Sigma). A mixture of *in vitro* transcribed sgRNA (10 ng/μl) and *Cas9* mRNA (40 ng/μl) was injected into the cytoplasm of pronuclear stage embryos. The injected embryos were transferred to embryo culture medium and kept for 30 min at 38.5°C, with 5% CO_2_ and humidity conditions. Then the embryos were transferred into the oviduct of the recipient mother.

### Genotyping of MSTN mutations

Genomic DNA was extracted from the blood of F0 and F1 MSTN mutation goats. Sanger sequencing after PCR amplification identified *MSTN* gene mutations. Primers used in PCR amplification were: 5′-TAACTCTTCTTTCCTTTCC3′ (forward) and 5′-CTGAAGTGGTACCTAATTTC-3′ (reverse).

### Off-target analysis

The POTS of the two sgRNAs were predicted using the CRISPR design tool (http://crispr.dbcls.jp/). POTS with top three ranking scores were selected for sgRNA1 according to ranking scores. Primers used in the present study are shown in Table 2. PCR products were subjected to Sanger sequencing. Goat genome has no sequence conformation to the potential Off-target site with sgRNA-2. See Table 2 for details.

**Table 2:** POTS and primer sequences for sgRNA1

POTS	Sequence	Primer sequences	Product length
POST1	5′-ATCTTTGAAGGAGTACAGCCTAG-3′	5′-ATAAACCCTAACTTCCACC-3′ (forward) and 5′-CCTACTCCACCCTACTCAC-3′ (reverse)	477
POST2	5′-CTCGTTGTAGGGGTACAGCAAGG-3′	5′-TTAGGGGTAAACTGGAGA-3′ (forward) and 5′-AGTAGCCTGACTGGAAAA-3′ (reverse)	448
POST3	5′-AATTTTGTTGGAGAACAGCAAGG-3′	5′-CTCCAGCATCCATACC-3′ (forward) and 5′-CATCTTCCTTCAACCC-3′ (reverse)	473

### Histology analysis

Tissues of gluteus maximus from MSTN knocked-out (KO) and wild-type (WT) goats of F0 and F1 generation were collected at the birthday (1 year) by a minimal invasive procedure to make tissue sections. Tissues were subjected to H&E staining and photographed at 200× magnification under a microscope (ts100, Nikon, Japan). Muscle cross-sectional area was calculated using ImageJ software (National Institutes of Health, New York, U.S.A.). Data for *myo*-fibers were analyzed by a general linear model of regression using SPSS (General Linear Model, SPSS 11.0; SPSS, Inc., U.S.A.).

### Body weight

MSTN KO goats and 12 WT (six male goats and six female goats with the same age) goats were raised under the same conditions. Body weights were recorded at days 0, 20, 36, 60, 90, and 210, respectively.

### Analysis of biochemical constituents to study the nutrition metabolism

Blood sample was collected from the jugular vein at days 36, 60, 90, and 210, respectively to prepare serum sample. Levels of alanine aminotransferase (ALT), aspartate aminotransferase (AST), total protein (TP), albumin (ALB), glucose (GLU), blood urea nitrogen (BUN), creatinine (CREA), triglyceride (TG), total cholesterol (TC), high-density lipoprotein cholesterol (HDL-C), low-density lipoprotein cholesterol (LDL-C), calcium (CA), phosphorus (P), uric acid (UA), lipase (LIP), pancreatic amylase activity (PAMY), ALP (alkaline phosphatase) in serum were measured using a blood biochemical analyzer (UniCel DxC 800 Synchron, Beckman Coulter).

### Genetic stability and reproductive ability analysis

Sperms from three male MSTN mutation goats were collected artificially to evaluate the genetic stability by PCR. Male MSTN mutation goats were mated with four normal goats, respectively, to analyze the reproductive ability.

### Statistical analysis

Data were statistically analyzed using GraphPad Prism software (*t* test) and a *P*-value <0.05 was considered to be statistically significant.

## Results

### Generation of MSTN KO goats by CRISPR/Cas9 system

Six surrogate mothers gave birth to eight live pups successfully. T-cloning and PCR-sequence results showed that the MSTN mutation was detected in six live pups (two MSTN^−/−^ and four MSTN^+/−^), named M1, M1, M3, M4, M5, and M6. The indels ranged from 2 to 13 bp. In addition, fragment deletions between the two sgRNAs targetting sites were frequently observed in the present study (100%, see Supplementary Material for details). Furthermore, the typical phenotype of double muscle (compared with their WT counterparts) was observed in varying degrees in F0 MSTN KO goats. PCR results showed that no mutation was detected in these POTS, indicating that the Cas9 sgRNA system did not induce undesirable off-target effect in MSTN KO goats.

### Comparison of body weight and phenotypes

**Figure 1:**
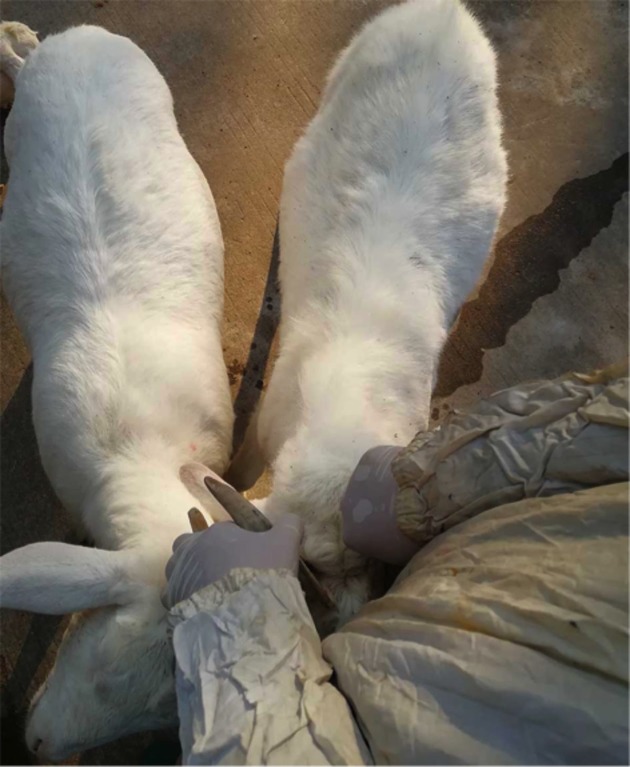
Comparison of phenotype at 100 days after birth The left goat is MSTN^−/−^ goat (M1) and the right one is WT goat. Notice the differences in muscle from the posterior, waist muscle and shoulder muscle.

As shown in Table 3, no significant differences in body weight were found between MSTN KO goats and WT goat at days 0, 20, 36, 60, 90, and 120. However, body weight of MSTN KO goats at day 210 was significantly higher than that of WT goats at day 240 (*P*<0.05). In addition, the average body weight gain (BWG) was also significantly higher in MSTN KO goats than in WT goats. As shown in Figure 1, the left goat is MSTN^−/−^ goat (M1) and the right one is WT goat. Muscle from the posterior, waist muscle and shoulder muscle of MSTN^−/−^ goat were obviously bigger than those of WT goats.

**Table 3:** Comparison of body weight at multiple time points

	0	20th day	36th day	60th day	90th day	120th day	210th day	Gain (kg)/day
M2	3.25	7.19	9.95	13.70	17.60	21.20	32.38	0.18
M4	4.05	7.39	10.21	13.17	20.50	23.30	36.74	0.16
MSTN KO	3.25 ± 0.94	7.09 ± 1.30	9.63 ± 2.06	13.07 ± 2.22	17.32 ± 2.62	20.31 ± 3.48	31.21 ± 4.09	0.13
WT	3.36 ± 0.48	7.60 ± 1.25	10.46 ± 1.07	13.69 ± 1.65	15.17 ± 2.35	17.35 ± 1.91	25.09 ± 2.33*	0.11

*The body weight of MSTN KO goats at day 210 was significantly higher than that of WT goats at day 240 (*P*<0.05).

### Hyperplasia and/or hypertrophy of muscle fibers in MSTN KO goats

**Figure 2:**
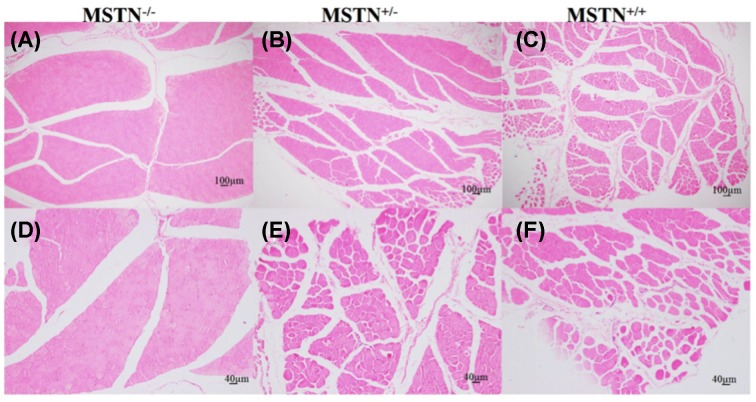
H&E staining of the muscle fibers from gluteus maximus

To determine whether the increase in muscle mass is due to hyperplasia and/or hypertrophy of muscle fibers, histological analysis was performed using gluteus maximus obtained from WT and MSTN KO goats. Results showed that the average size of gluteus maximus was significantly increased in the MSTN^−/−^ (980 µm^2^) goats than in WT goats (719.76 s µm^2^, *P*<0.01). Furthermore, the average fiber density in gluteus maximus from MSTN^−/−^ goats (2500s N/mm^2^) was significantly (*P*<0.01) higher than that in WT goats (900 N/mm^2^) (Figure 2). These results therefore suggest that the increased muscle mass of gluteus maximus in the MSTN KO goats is due to both fiber hyperplasia and hypertrophy.

### Evaluation of health status of goats with MSTN mutation

In order to investigate the effect of targetted MSTN mutation on nutrition metabolism in goats, multiple biochemical parameters were measured at days 36, 90, and 210. As shown in Table 4, no significant differences in serum level of ALT, AST, GGT, TBIL, DBIL, LDH, CK, and GLO were found between MSTN KO goats and WT goats at different time points. Serum ALP levels in MSTN KO goats were significantly higher than those in WT goat at days 36, 90, and 210 (*P*<0.05). Serum levels of BUN in MSTN KO goats were significantly lower than those in WT goat at day 90 (*P*<0.05). Serum CREA levels in MSTN KO goats were significantly higher than those in WT goat at days 36, 90, and 210 (*P*<0.05). Levels of Ca of MSTN KO goats were significantly higher than those of WT goats at day 90 (*P*<0.05). Levels of HDL-C were significantly higher in MSTN KO goats than in WT goats at days 36, 90, and 210 (*P*<0.05). No significant differences in serum level of CHOL, TG, and LDL-C were found between MSTN KO goats and WT goats at different time points. Those results suggest MSTN plays an important role in metabolic regulation of sugar, fat, and protein in goats.

**Table 4:** Biochemical constituents in MSTN KO goats and WT goats

	Day 36th		Day 90th		Day 210th	
	MSTN^+/+^	MSTN KO	MSTN^+/+^	MSTN KO	MSTN^+/+^	MSTN KO
ALT(U/l)	17.80 ± 3.06	16.18 ± 2.14	18.22 ± 4.09	17.87 ± 1.83	23.04 ± 2.35	24.47 ± 3.16
AST (U/l)	70.82 ± 11.67	75.28 ± 7.43	76.73 ± 10.78	73.67 ± 4.31	96.62 ± 15.07	93.87 ± 3.55
GGT (U/l)	43.83 ± 4.62	44.50 ± 2.81	42.00 ± 4.00	39.00 ± 2.61	42.33 ± 2.42	39.67 ± 1.63
TBIL (μmol/l)	4.32 ± 0.58	4.15 ± 0.26	4.50 ± 1.69	4.65 ± 1.34	7.73 ± 1.22	7.50 ± 0.47
DBIL(μmol/l)	2.07 ± 0.37	2.08 ± 0.26	2.65 ± 0.15	2.56 ± 0.10	2.60 ± 0.45	2.43 ± 0.28
LDH (U/l)	346.33 ± 20.96	349.17 ± 30.01	324.33 ± 32.54	339.33 ± 25.31	347.83 ± 19.24	357.50 ± 28.65
CK (U/l)	165.67 ± 37.43	166.17 ± 22.83	200.33 ± 41.23	179.00 ± 18.93	280.67 ± 35.39	317.33 ± 16.42
GLO (g/l)	27.82 ± 2.76	28.30 ± 4.55	42.45 ± 5.29	37.70 ± 4.55	44.37 ± 3.82	47.02 ± 3.19
TP (g/l)	55.47 ± 3.80	55.18 ± 3.73	70.55 ± 11.13	70.18 ± 6.95	76.95 ± 7.02	79.38 ± 2.98
ALB (g/l)	27.65 ± 1.96	26.88 ± 2.16	28.10 ± 7.96	32.48 ± 2.25	31.17 ± 1.16	32.37 ± 3.76
BUN (mmol/l)	4.71 ± 0.55	4.11 ± 0.69	11.42 ± 2.38	7.51 ± 2.39	10.91 ± 3.87	8.86 ± 2.93
UA (μmol/l)	36.93 ± 1.16	35.35 ± 1.86	22.55 ± 1.51	22.35 ± 1.95	21.50 ± 3.22	19.02 ± 2.72
CREA (μmol/l)	47.67 ± 6.03	80.23 ± 20.38	33.45 ± 5.88	53.48 ± 10.66	38.58 ± 5.90	53.15 ± 6.65
Ca (mmol/l)	2.36 ± 0.29	3.21 ± 0.51	2.55 ± 0.20	3.08 ± 0.56	2.45 ± 0.40	2.83 ± 0.42
P (mmol/l)	2.99 ± 0.27	2.98 ± 0.30	2.22 ± 0.52	2.70 ± 0.32	2.80 ± 0.39	2.69 ± 0.51
ALP (mmol/l)	260.83 ± 25.95	321.17 ± 53.04	86.67 ± 14.07	167.33 ± 26.52	199.84 ± 17.95	246.50 ± 41.25
GLU (mmol/l)	2.57 ± 0.27	2.33 ± 0.23	2.93 ± 0.33	2.25 ± 0.36	2.81 ± 0.13	2.23 ± 0.39
PAMY (U/l)	16.50 ± 3.45	17.67 ± 3.61	17.33 ± 6.19	19.67 ± 3.83	22.83 ± 5.15	22.50 ± 5.24
CHOL (mmol/l)	4.00 ± 1.20	4.99 ± 1.15	2.26 ± 0.55	2.56 ± 0.29	1.99 ± 0.41	2.35 ± 0.47
TG (mmol/l)	0.60 ± 0.16	0.75 ± 0.21	0.64 ± 0.12	0.79 ± 0.33	0.71 ± 0.11	0.75 ± 0.09
HDL-C (mmol/l)	1.07 ± 0.17	1.49 ± 0.25	0.90 ± 0.32	1.54 ± 0.37	0.86 ± 0.10	1.35 ± 0.22
LDL-C (mmol/l)	0.91 ± 0.06	1.07 ± 0.19	0.83 ± 0.34	1.11 ± 0.18	0.69 ± 0.11	0.84 ± 0.13
LIP (U/l)	16.93 ± 2.83	15.45 ± 0.86	31.88 ± 6.85	35.08 ± 3.48	36.92 ± 4.82	36.77 ± 4.18

Abbreviations: ALT, alanine aminotransferase; AST, aspartate aminotransferase; CHOL, cholesterol; CK, creatine kinase; DBIL, direct bilirubin; GGT, γ-glutamyl transpeptidase; GLO, globulin; LDH, lactate dehydrogenase; LIP, lipase; TBIL, total bilirubin.

### Heritability and reproductive ability of the MSTN KO goats

**Figure 3:**
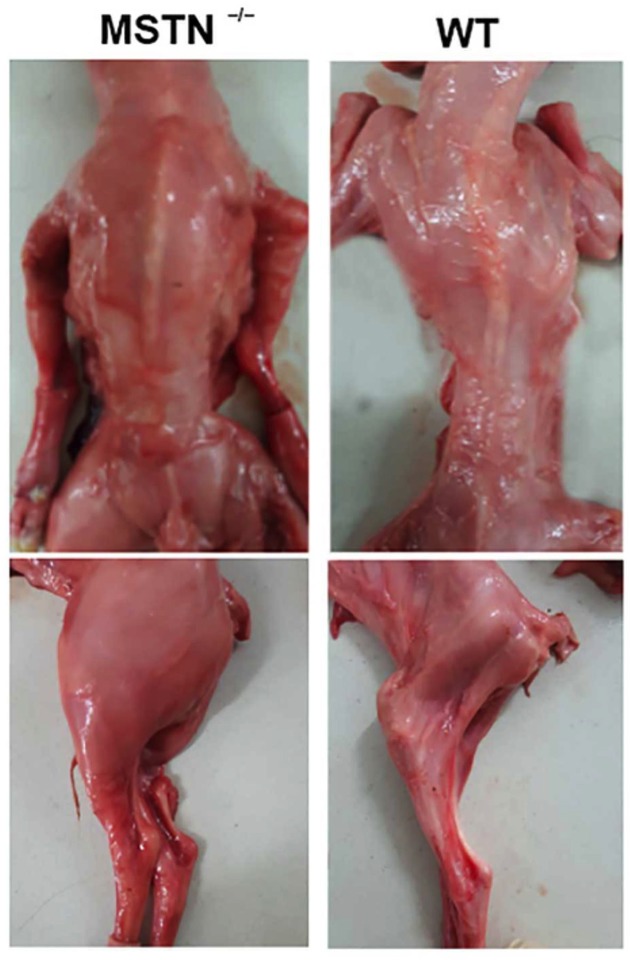
Comparison of phenotype of offspring of MSTN^−/−^ goat (M1) and WT goat The left is the offspring of MSTN^−/−^ goat, the right is the offspring of WT goat. Notice the differences in muscles at different positions.

To determine whether the MSTN KO can be stably transmitted to the offspring, *MSTN* gene sequences of offspring were amplified by PCR and sequenced by Sanger sequencing. Results showed that two out of three newborn F1 goats were with MSTN mutations. All of the 12 normal goats that mated with MSTN KO F0 male goats were pregnant. Offspring was obtained through cesarean section. As shown in Figure 3, offspring of MSTN KO goat (M1) maintained the phenotype.

## Discussion

At present, generation of MSTN mutations has been widely used to study muscle development and increase meat production in various animal species, such as rabbit [8], sheep [9], and goat [10]. However, as far as we know, no ideal targetted phenotypes have been observed in goats with MSTN mutation. In the present study, we successfully generated MSTN KO goats by microinjection of Cas9/sgRNA into zygotes, and deletions were frequently detected between two sgRNAs targetting sites in MSTN KO goats, suggesting that the dual sgRNA-directed CRISPR/Cas9 system is an efficient tool for gene knockout in mammal genome.

In the present study, two MSTN^−/−^ goats showed very different phenotypes, and typical double-muscled (DM) phenotype was only found in M2. In M2, 35 amino acids in one chain and 36 amino acids in the other were mutated. The other MSTN^−/−^ goat showed similar phenotype to that of MSTN^+/−^ goats, although its growth rate was higher than that of WT goat, but the double muscle phenotype was not satisfactory. Kambadur et al. [11] reported the destroyed MSTN EXON 3 cysteines knots in Belgian Blue cattle made the double muscle phenotype. Based on previous studies and the findings in our study, we concluded that targetting site and the number of mutated amino acids determine DM phenotype. In addition, in our study, sgRNA1 successfully targetted MSTN in five goats, while targetting of MSTN by sgRNA2 was only observed in one MSTN KO goat. Therefore, targetting sites also determine targetting efficiency.

BUN in serum is a sensitive marker to reflect protein intake in the body, and lower BUN level predicts lower protein intake rate [12]. In this study, serum levels of BUN were found to be significantly higher in MSTN KO goats than in WT goats, which may be an explanation of higher BWG rate in MSTN KO goats than in WT goats. Previous studies have showed that MSTN is a key player in the pathways involved in fat metabolism [13,14], and inhibition of MSTN expression can improve obesity by enhancing fatty acid oxidation and promoting the formation of brown adipose in mice [15]. No significant differences were found in serum UA between mutants and WT, indicating the normal renal function of mutants. CA level was significantly higher in MSTN KO goats than in WT goats, which indicates the increased bone density of MSTN KO goats. CREA is essential for muscle and bone development [16]. Higher serum CREA levels in MSTN KO goats suggest the better muscle and bone conditions compared with WT goats. To our surprise, serum level of CHOL, HDL-C, and LDL-C were all significantly higher in MSTN KO goats than in WT goats, which is inconsistent with the findings in a previous study [10]. The possible explanation may include the existence of different nutrition metabolism regulation pathways at different ages and the different effects of different MSTN mutations on fat metabolism.

Off-target mutation has been frequently reported in Cas9-mediated gene editing system [17,18], and numerous efforts have been made to reduce the off-target rate [19–21]. Our future study will try to carry out a whole gene sequencing to detect the off-target effect in the present study. Relatively low concentration of Cas9/sgRNA was used in the present study, and Cas9/sgRNA may be degraded immediately after targetting of MSTN. In addition, the strict match of seed sequences (8–12 bases close to PAM) in our study may be another reason for the site-specific cleavage of CRISPR/Cas9 system.

It has been demonstrated that the MSTN-deficient animals may show disorders similar to large offspring syndrome [22]. In our study, a dramatically increased body weight and typical DM phenotypes were observed, body weight of MSTN KO goats were not significantly different from that of WT goat at birth. In addition, reproduction problems were not observed. These data suggest that MSTN KO goats generated by CRISPR/Cas9 system can provide reliable data for different purposes.

## Conclusion

In conclusion, MSTN KO goats were successfully generated by targetting the exon3 of MSTN using CRISPR/Cas9 system. The average BWG per day of MSTN KO goats was significant higher than that of WT goats. MSTN KO goats showed abnormal sugar, fat, and protein metabolism compared with wild-type controls (MSTN^+/+^). Offspring of MSTN KO goats showed high genetic stability and reproductive ability.

## Supporting information

**Table:** 

## Abbreviations

ALP: alkaline phosphatase
ALT: alanine aminotransferase
AST: aspartate aminotransferase
BUN: blood urea nitrogen
BWG: body weight gain
CA/Ca: calcium
Cas9: CRISPR-associated protein 9
CREA: creatinine
CRISPR: clustered regularly interspaced short palindromic repeats
DM: double-muscled
FGF5: fibroblast growth factor 5
HDL-C: high-density lipoprotein cholesterol
KO: knocked-out
LDL-C: low-density lipoprotein cholesterol
MSTN: myostatin gene
POTS: putative off-target sites
TG: triglyceride
sgRNA: guide RNA
UA: uric acid
WT: wild-type
