# Supplementary material for: Use of CRISPR/Cas9 technology efficiently targetted goat myostatin through zygotes microinjection resulting in double-muscled phenotype in goats
Source: Biosci Rep. 2018 Nov 14;38(6):BSR20180742. doi: 10.1042/BSR20180742 (PMC6239268; doi:10.1042/BSR20180742)
Supplement: Supplementary file 1 [file bsr20180742_Supp1.pdf]

Animal	SgRNA-2 PAM	Indels	TA	Amino acids
WT	AGGCATGGTAGTAGATAGTAATATGTGGGTGCTCA	WT		VVDRCGCS
M1	AGGCATGGTAGTAGATAGTAATATGTGGGTGCTCA	WT	X2	VVDRCGCS
	AGGCATGGTAGTAGATCGC-------TGTGGGTGCTCA	sub 2bp del5 bp	X3	VVDRXXWVLM

Animal	SgRNA-1 PAM	Indels	TA	Amino acids
WT	ACATCTTTGTAGGAGTACAGCAAGGGCCGGCTGAAC	WT		KGSAGPCCTPTKMSPINMLYFNGK
M2	ACATCTTTGTAGGAG----------CAAGGGCCGGCTGAAC	del 5bp	X3	KGSAGPCSYKDVSN.Y
	ACATCTTTGTAGGAGTACA----AAGGGCCGGCTGAAC	del 2 bp	X3	KGSAGPXCSYKDVSN.YA
M3	ACATCTTTGTAGGAGTACAGCAAGGGCCGGCTGAAC	WT	X3	KGSAGPCCTPTKMSPINMLYFNGK
	ACATCTTTGTAGG------------------AGGGCCGGCTGAAC	del 9 bp	X3	KGSAGP---PTKMSP
M4	ACATCTTTGTAGGAGTACA------AGGGCCGGCTGAAC	del 3 bp	X3	KGSAGP-CTPTKMSPINMLYFNGK
	ACATCTTTGTAGGAGTACA--------------CC-------------AC	del 13bp	X3	KG--G--VLLQRCLQLICYILMAKNK.YM
M5	ACATCTTTGTAGGAGTACAGCAAGGGCCGGCTGAAC	WT	X3	KGSAGPCCTPTKMSPINMLYFNGK
	ACATCTTTGTAGGAGTACA------AGGGCCGGCTGAAC	del 3 bp	X3	KGSAGP---PTKMSP
M6	ACATCTTTGTAGGAGTACAGCAAGGGCCGGCTGAAC	WT	X3	KGSAGPCCTPTKMSPINMLYFNGK
	ACATCTTTG---------------------CAAGGGCCGGCTGAAC	del 11 bp	X3	KGSAGPCKDVSN

Notes Sub: substitution; del: deletion
